# Construct validation of the Research Engagement Survey Tool (REST)

**DOI:** 10.1186/s40900-022-00360-y

**Published:** 2022-06-16

**Authors:** Melody S. Goodman, Nicole Ackermann, Zoé Haskell-Craig, Sherrill Jackson, Deborah J. Bowen, Vetta L. Sanders Thompson

**Affiliations:** 1 New York University School of Global Public Health, New York, USA; 2 Washington University in St. Louis, St. Louis, USA; 3 The Breakfast Club, Inc., Florissant, USA; 4 University of Washington, Seattle, USA

**Keywords:** Research engagement, Stakeholder engagement, Validation, Survey measure, Construct validation, Convergent validity, Internal consistency

## Abstract

**Background:**

The Research Engagement Survey Tool (REST) was developed to examine the level of partner (e.g., patients, caregivers, advocates, clinicians, community members) engagement in research studies. The REST is aligned with eight engagement principles based on the literature and consensus reached through a five round Delphi process. Each of the engagement principles has three-five corresponding items that are assessed on two Likert type scales quantity (how often: never, rarely, sometimes, often, always, not applicable) and quality (how well: poor, fair, good, very good, excellent, not applicable). We conducted a comprehensive validation of the REST. Despite the importance of partner engagement in research, currently no gold standard measure exists.

**Methods:**

Multiple strategies were employed to validate the REST. Here, we examine the internal consistency of items for each of the eight engagement principles. In addition, we examine the convergent validity of the comprehensive (32-item) REST with other measures (e.g., medical mistrust, Community Engagement in Research Index, Partnership Self-Assessment Tool, Wilder collaboration inventory, Partnership Assessment In community-based Research). We propose two scoring approaches for the REST; one aligned with the engagement principles and the other aligned with levels of community engagement: (1) outreach and education, (2) consultation, (3) cooperation, (4) collaboration, and (5) partnership.

**Results:**

The REST has strong internal consistency (Cronbach’s alpha > 0.75) for each of the eight engagement principals measured on both scales (quality and quantity). The REST had negligible (e.g., medical mistrust, community engagement in research index), low (e.g., Partnership Assessment In community-based Research, Partnership Self-Assessment Tool- benefits scale), and moderate (e.g., Wilder collaboration inventory, Partnership Self-Assessment Tool- synergy scale) statistically significant correlations with other measures based on the Spearman rank correlation coefficient. These results suggest the REST is measuring something similar and correlated to the existing measures, but it captures a different construct (perceived research engagement).

**Conclusions:**

The REST is a valid and reliable tool to assess research engagement of community health stakeholders in the research process. Valid tools to assess research engagement are necessary to examine the impact of engagement on the scientific process and scientific discovery and move the field of stakeholder engagement from best practices and lessons learned to evidence-based approaches based on empirical data.

**Supplementary Information:**

The online version contains supplementary material available at 10.1186/s40900-022-00360-y.

## Plain English summary

Researchers often conduct studies with partners (e.g., patients, caregivers, advocates, clinicians, community members) who also have an interest in the research topic. Depending on the research study the level of partner engagement in the research process may be high or low. Partners may be involved from the beginning including determining what topic to study and what questions the study should examine. They may suggest who should be included in the study, the geographic area of focus, and the outcome measures to be examined. In addition, they may help recruit study participants, interpret study results, and plan for how to share the results with those that need to know. No standard way exists to find out how involved a partner has been in a study from the partner’s perspective. Here we develop and validate survey questions to measure the level of partner engagement in research studies. We looked at existing survey questions used to measure similar topics to make sure that a person who takes the survey gets consistent scores. We tested the survey with community health stakeholders (e.g., patients, caregivers, advocates, clinicians, community members) who are research partners for studies at universities across the United States. Over 2 years, the partners took different versions of the survey online four times. We used the data we collected from each survey to revise the questions and make sure that it measures partner involvement accurately and reliably. The Research Engagement Survey Tool (REST) has 32 questions to examine eight engagement principles on two scales: quantity (how much) and quality (how well). The REST is a valid and reliable tool to examine partner engagement in research.

**Supplementary Information:**

The online version contains supplementary material available at 10.1186/s40900-022-00360-y.

## Background

Stakeholder engagement in research is the process of ensuring that key community health constituents are identified and involved throughout the research process as partners (investigators not participants). Ideally this involvement starts before project inception, so that they are able to inform study design, implementation, interpretation of results, and make use of the results when the study is completed [1]. There has been a call for better reporting and evaluation of engagement approaches, initiatives, and activities to advance the science of stakeholder engagement [2]. The engagement of stakeholders (e.g., patients and their families, clinicians, health systems, policy makers, community organizations, advocacy groups) in research projects has created lessons learned and best practices. However, few methods exist for measuring the extent to which stakeholders are engaged in a research project (e.g., quality of engagement efforts), limiting the ability to determine evidence-based approaches for stakeholder engagement [2]. This poses two major problems to advancing stakeholder engaged research. The first is that it is difficult to compare the effectiveness of various strategies employed by different research teams in incorporating stakeholder views and input. The second problem lies in determining the effect of stakeholder engaged research practices on the rates of program adoption and success of implementation.

Currently, researchers must work from a set of case studies and ‘best practices’ recommendations (e.g., actively seeking collaboration with diverse populations, offering many opportunities to give input in a variety of formats and venues, going to where people are, being transparent and trustworthy). For instance, Holzer et al. use three case studies to demonstrate some key elements (e.g., building trust, encouraging participation, promoting uptake of findings) of successful approaches to community engagement in research [3]. However, the breadth of disciplines that undertake stakeholder engaged research impedes any kind of generalization of best practices. Furthermore, stakeholder engagement can occur at any stage of research, yet may look very different in the early stages of a research project (such as hypothesis development) as compared to the dissemination phase of a translational research project. It is impossible to gauge from the existing literature what level of engagement is necessary for a study and what types of engagement practices would be best given a particular population and research question.

Reviewers of the literature tend to suggest community engagement practices have some positive impact on health improvement interventions for a range of health outcomes across various conditions. However, there is insufficient evidence to determine whether one particular model of community engagement is more effective than any other [4, 5]. In addition, such articles also simultaneously note substantial variation in the effectiveness of different practices on improving interventions without being able to determine whether any one approach consistently outshines the rest [6, 7]. A systematic review found no evidence of impact from community engagement on population health or the quality of services, but engagement initiatives did have positive impacts on housing, crime, social capital, and community empowerment. Methodological developments are needed to enable studies of complex social interventions to provide robust evidence of population impact in relation to community engagement. With no consistent approach to measuring engagement, conducting analyses across multiple studies is ineffectual.

Current approaches to measuring stakeholder engagement focus largely on qualitative methods [8–12]. Despite their efficacy at assessing engagement, these methods are difficult to scale up for large-scale projects and produce results that are difficult to compare across studies and do not generalize well into standard practices [13]. For these reasons Bowen et al. called for the development of a quantitative scale, grounded in theory, that is comprehensive in evaluating all elements of engagement, is easy to use, and provides psychometric data [14]. Such a scale, the *Research Engagement Survey Tool (REST)*, has been proposed [15, 16], and has been comprehensively evaluated. This paper examines the internal consistency (reliability) and convergent validity of the REST.

The original version of REST was developed by the evaluation team of the Program for the Elimination of Cancer Disparities (PECaD) at Siteman Cancer Center [17, 18] and pilot tested in one of its programs [13]. The original version of the REST was designed to align with 11 engagement principles selected by the PECaD’s community advisory board (Disparities Elimination Advisory Committee) based on the community based participatory research and community engagement literature [11, 19–29]. Subsequently, revisions to the measure have been made through a five round Delphi process [15, 16, 30] and cognitive response testing [31]. The final version examines eight engagement principles (EPs) [16], applicable along the full continuum of engagement activities [15]. The EPs are:

1. Focus on community perspectives and determinants of health
2. Partner input is vital
3. Partnership sustainability to meet goals and objectives
4. Foster co-learning, capacity building, and co-benefit for all partners
5. Build on strengths and resources within the community or patient population
6. Facilitate collaborative, equitable partnerships
7. Involve all partners in the dissemination process
8. Build and maintain trust in the partnership

Each EP is assessed using three to five items that were measured on two scales: quality (how well: poor, fair, good, very good, excellent, not applicable) and quantity (how often: never, rarely, sometimes, often, always, not applicable) with five-point Likert response options. The stem for the quantity scale is “Please rate how often the partners leading the research do each of the following” and for the quality scale the question stem is “Please rate how well the partners leading the research do each of the following”. There are measures for key constituent stakeholder groups (e.g., patients [32], community [33], community advisory boards [34], coalitions [35]), however, the REST is unique in that it is applicable to all non-academic research partners and is based on their perspective.

Ideally, such a proposed psychometric scale has both high reliability—records consistently the same values for research project stakeholder engagement independent of who is reviewing—and high validity—that is, draws accurate conclusions about the presence and degree of stakeholder engagement [36]. Cronbach’s alpha is a well-developed measure of reliability, characterizing how strongly the items within each EP resemble each other [37], and is robust to the sample size of surveys conducted [38]. Nunnally and Bernstein propose a value of alpha = 0.80 as a satisfactory level of reliability, beyond which decreasing measurement error has little effect on the value for alpha [39]. Ideally, the validity of a scale would be evaluated by demonstrating that the tool produces results that agree with the ‘gold standard’ test. No gold standard exists for measuring stakeholder engagement, therefore convergent validity (the degree to which two measures of constructs that theoretically should be related, are in fact related) was used to assess construct validity by comparing the results from REST to a number of other scales that similarly measure engagement (e.g., Partnership Assessment In community-based Research [40]). Included in this comparison are also tools for measuring constructs that are theoretically associated with strong stakeholder engagement (e.g., trust in medical researchers and health literacy). This paper presents the analysis of reliability and construct validity of the REST measure.

## Methods

### Study overview

**Fig. 1:**
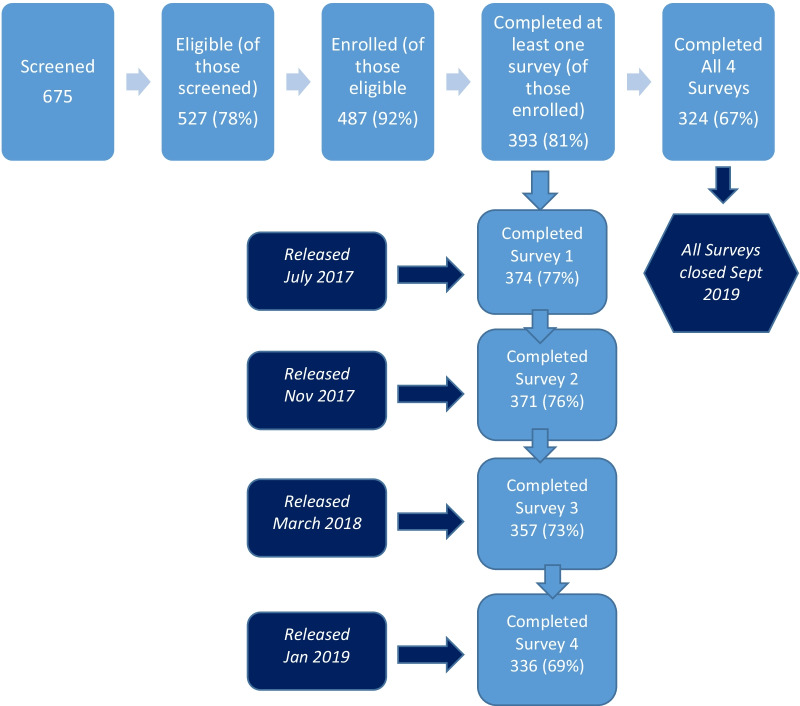
Participants complete a short screening instrument. Those screened eligible are sent a link to the survey. Participants that complete the informed consent screen by agreeing to participate are considered enrolled. Surveys were open from July 2017 (depending on release date) to September 2019

The study was composed of four longitudinal web-based surveys conducted between July 2017 and September 2019 (see Fig. 1). The modified versions of REST presented on sequential surveys correspond to the versions revised through a Delphi process described in detail elsewhere [16, 30]. Surveys one through three contained measures that assess dimensions of collaboration, partnership, trust in medical researchers, and engagement used in determining convergent validity. Finally, we released the fourth survey in January 2019 and it contained the final version of the REST [16, 30, 31] and asked participants to review the categories of community engagement in research and their corresponding definitions [15] and to classify their project into one of these categories: (1) outreach and education, (2) consultation, (3) cooperation, (4) collaboration, and (5) partnership.

### Participants

We recruited participants (community partners in research studies) through several different methods throughout the study period (July 2017 to August 2019). Our first recruitment approach consisted of email recruitment to principal investigators (PIs) in the research team’s network involved with stakeholder-engaged research and contacts in health departments, Clinical and Translational Science Awards (CTSA) Programs, Prevention Research Centers, Transdisciplinary Research in Energetics and Cancer Centers, National Institute on Minority Health and Health Disparities Centers of Excellence, National Cancer Institute Community Networks Programs, and U.S. Department of Health & Human Services Regional Health Equity Councils. We also developed a database of community-engaged researchers nationally and reached out to them via email. We asked PIs of community engaged research studies to share information about this study with their community partners. To complement email recruitment, we conducted in person recruitment by attending local (St. Louis, MO) health fairs and local community partner meetings, posting recruitment flyers locally and attended national conferences related to community engagement. We also used recruitment resources at Washington University in St. Louis, including the Washington University Recruitment Enhancement Core and Research Match, a national health volunteer registry created by several academic institutions and supported by the National Institutes of Health as part of the CTSA program (https://www.researchmatch.org/).

Participants were included in the study if they were currently or previously involved in stakeholder-engaged research, were over age 18, and were willing to participate in an 18-month longitudinal study based on an electronic screening instrument (Additional file 1: Table S1). We removed participants from this study that screened eligible but completed the survey multiple times, provided invalid telephone numbers or zip codes, or had odd patterns in their responses (N = 95) [41, 42]. We screened 675 people, of whom 527 (78%) were eligible. Of those eligible, 487 (92%) enrolled in the study (completed informed consent). Of those enrolled, 393 (81%) completed at least one of the four surveys, while 324 (67%) completed all four surveys. See Fig. 1 for participant recruitment and survey timeline.

### Procedures

Potential participants completed an eligibility screener (Additional file 1: Table S1) answering questions pertaining to the inclusion and exclusion criteria described above either online or in person. In person screeners were completed either on paper or via a tablet with a member of the research team present. The vast majority of eligible participants provided an email address and were emailed a personalized link to the first survey within two business days. Participants recruited in person that did not have an email address were provided the first survey in person, completed, and returned to a member of the research team (n = 8). After completion of the first survey, participants were provided with a $10 gift card.

For participants completing the surveys online, surveys two through four were emailed to participants either on the survey release date, or if the participant enrolled in the study after the initial survey release date, the survey was emailed to participants within five business days of completing the previous survey. When new surveys were released, they were sent to all enrolled participants, regardless of completion status for previous surveys. Participants who enrolled after initial survey release dates were sent a link to the subsequent survey within approximately four weeks of being sent the previous survey if they had not yet completed the previous survey. All online surveys, including the eligibility screener, were administered through the survey platform Qualtrics (Provo, UT).

For participants completing the surveys on paper, a member of the research team brought participants the subsequent surveys during meetings they both attended and the participants completed the surveys and returned them to the research team. Participants received $10 for completing survey two, and an extra $5 if they completed both surveys one & two. Participants received $15 per survey for completing surveys three and four, and an extra $10 if they completed both surveys. The Institutional Review Boards at both Washington University in St. Louis and New York University approved all portions of this project.

### Measures

#### Research Engagement Survey Tool (REST)

The original version of REST was developed and pilot tested by the evaluation team for the Program for the Elimination of Cancer Disparities at Siteman Cancer Center [13, 17, 18]. The original version (survey one) contained 48 items corresponding to 11 engagement principles (EPs). Each EP contained three to five items that were measured on two scales: quality (how well) and quantity (how often). For the quality scale, response options were Poor, Fair, Good, Very Good, Excellent. For the quantity scale, response options were Never, Rarely, Sometimes, Often, Always.

Three additional revised versions of REST were presented sequentially on surveys two through four. Revisions were made based on a modified Delphi Panel process and cognitive interviews that have been described in detail elsewhere [16, 30, 31]. On survey four, an additional response option of ‘Not Applicable’ was added based on feedback from a Delphi panel process and cognitive interviews described elsewhere [16, 31].

#### Scoring REST

The REST has two scoring approaches, the first is aligned with EPs. We treat not applicable responses as missing in the analysis. REST scoring is done at the EP level and overall. EP specific scores were calculated as an average of non-missing items and the eight means were averaged to calculate the overall REST score. This scoring approach is used to examine the internal consistency and convergent validity of the REST. The second scoring approach aligns the REST with the categories of community engagement in research and provides a percentage in each of five categories: (1) outreach and education, (2) consultation, (3) cooperation, (4) collaboration, and (5) partnership [15]. This scoring approach does not provide one overall score, rather it is five percentages (one for each engagement level) based on the number of REST items (out of 32 total) that are scored in each category (using the scoring scheme provided in Additional file 1: Table S2) based on the survey responses.

To develop the second scoring approach, we reviewed each item against the definitions of the categories of engagement and identified the category for each response (Additional file 1: Table S2). For example, for item 1.4, “The focus is on cultural factors that influence health behaviors,” we classified it as follows:

- For quality: poor = outreach & education, fair = outreach & education, good = outreach & education, very good = consultation, excellent = cooperation. This means if a participant responds with poor, fair, or good one point is added to the outreach & education category, if the participant instead responds very good one point is added to the consultation category, or if the participant had responded excellent one point would be added to the cooperation category.
- For quantity: never = outreach & education, rarely = outreach & education, sometimes = outreach & education, often = consultation, excellent = consultation. This means if a participant responds never, rarely, or sometimes to item 1.4 one point is added to the outreach & education category, if the participant had instead responded often, one point would be added to the consultation category, or if the responded reported excellent for item 1.4 one point would be added to the consultation category.

A similar process was done for each item (Additional file 1: Table S2). To calculate the overall score by survey respondent, we gave each participant a point for the category of engagement based on their response for that item (Additional file 1: Table S2). For example, if a participant responded good for item 1.4 on the quality scale, they would get one point in the outreach & education category. Then, for each survey respondent, we summed the points for each category of engagement and calculated a percentage with 32 as the denominator; the version of REST that the participants completed had 32 items (comprehensive version). We examine the average percentages by category of engagement.

#### Other measures

On survey one, we included measures of health literacy [43, 44], subjective numeracy [45], medical mistrust [46], trust in medical researchers [47], a survey of community engagement [34], and the Partnership Assessment In community-based Research (PAIR) [40]. The measure of medical mistrust [46] was calculated as an unweighted sum score with 12 subtracted from the total. The trust in medical researchers score [47] was calculated as a percentage of the total range. For both the medical mistrust and trust in medical researchers scores, higher values indicate more trust in medical researchers. The Kagan et al. [34] summary score was calculated similar to REST, as a weighted average over the three sub-sections of community-involvement, relevance or research, and collaboration & communication. The Kagan et al. survey is a measure of the extent in which community advisory boards (CABs) are involved in research activities. The PAIR [40] measure was also calculated similarly, with a mean score for each dimension (communication, collaboration, evaluation/continuous improvement, benefits, and partnership) and then the dimension means averaged to create an overall score. The PAIR measure is designed to evaluate partnerships between community members and researchers. For both the Kagan et al. and PAIR measures, higher scores indicate higher engagement or a more developed partnership.

On survey two, we included the community engagement research index (CERI) [11], the trust subscale of the coalition self-assessment survey (CSAS) [35], and the community campus partnerships for health (CCPH) principles [48]. The CERI measures the level of community participation in research, while the trust subscale of the CSAS examines trust among coalition members and the CCPH principles measure collaborative partnerships between the community and academic institutions. The CERI was calculated according to Khodyakov et al. [11] by creating a summed index score over the 12 items with higher scores indicating more engagement in research. The trust portion of the CSAS was calculated as an average score [35], with higher values indicating higher trust.

On survey three, we included the partnership self-assessment tool (PSAT) [49, 50] and the Wilder collaboration inventory [51, 52]. The PSAT includes measures of 11 dimensions, including: (1) synergy, (2) leadership, (3) efficiency, (4) administration & management, (5) non-financial resources, (6) financial resources, (7) decision making, (8) benefits, (9) drawbacks, (10) comparing benefits and drawbacks, and (11) satisfaction. Each dimension has several items that are averaged together to create the overall dimension score, with higher scores indicating higher levels of the dimension, except for the benefits and drawbacks scales which are created as percentage scores and the comparison of benefits and drawbacks which consists of only one item [49, 50]. The Wilder collaboration inventory contains 40 total items pertaining to 20 factors of collaboration (one to three items per factor), within six overall categories of collaboration (environment, member characteristics, process/structure, communication, purpose, and resources; two to six factors per category) that are averaged to create an overall score [51, 52].

Demographic questions (age, gender, race, ethnicity, education level, region) and project description questions were presented on survey one, however, if a participant had not previously responded to survey one before they were sent the subsequent surveys, the demographic questions and project descriptions questions were asked of the participant on whichever survey they completed first. Age was measured continuously in years and gender was coded as male, female, or other. Race and ethnicity were asked as two separate questions but were combined into categories of Non-Hispanic/Latino(a) Black, Non-Hispanic/Latino(a) White, Hispanic, Asian, and Other/ Multiracial/ Unknown. Education level was coded as less than high school, high school degree or GED, some college or associates degree, college degree, or graduate degree. Region was coded as northeast, west, south, midwest, and non-state area (includes Virgin Islands and Puerto Rico). Project description questions included the following: open ended description of project and project purpose, the participants’ project role, how long participant had worked on the project, and how long the participant had collaborated with the academic/university partner.

### Statistical analysis

Descriptive statistics including mean, median, and standard deviation were calculated by item, EP, and for the overall measure. Frequencies and percentage of ‘not applicable’ responses by items were also calculated. We calculated Cronbach’s alpha for each EP of REST to assess internal consistency. To measure convergent validity of REST with other similar constructs, we calculated Spearman’s correlation coefficients between REST and other measures (i.e., Trust in Medical Researchers, Medical Mistrust, PAIR, CERI, Wilder Collaboration inventory, CSAS, PSAT, Kagan survey of community engagement). The addition of the ‘not applicable’ response option led to a larger number of missing responses on the version of REST presented on survey four. We therefore conducted sensitivity analysis throughout, using a sample of only those with all non-missing items and examined differences between the results. We conducted all aforementioned analyses for both the quality and quantity response scales of REST. All statistical analyses were conducted in SAS ® version 9.4.

## Results

The majority of participants were female (80%), from the Midwest region of the United States (53%) and had a college degree or higher level of education (75%). The participants were mostly either non-Hispanic/Latino(a) Black (41%) or non-Hispanic/Latino(a) White (42%) and had a mean age of 42 years (Table 1).

**Table 1:** Demographic characteristics of participants who enrolled (n = 487) and completed survey 4 (n = 336)

	Total enrolled N (%)	Completed survey 4 N (%)
*Race*		
Non-Hispanic/Latino(a) Black	201 (41.3%)	147 (43.8%)
Non-Hispanic/Latino(a) White	206 (42.3%)	140 (41.7%)
Hispanic	31 (6.4%)	17 (5.1%)
Asian	21 (4.3%)	16 (4.8%)
Other/multiracial/unknown	28 (5.8%)	16 (4.8%
*Gender*		
Male	92 (19.2%)	69 (20.5%)
Female	386 (80.4%)	267 (79.5%)
Other	9 (1.8%)	0 (0%)
*Education*		
Less than HS	5 (1.0%)	2 (0.6%)
HS degree or GED	17 (3.5%)	12 (3.6%)
Some college or Associate degree	98 (20.4%)	73 (21.7%)
College Degree	133 (27.7%)	97 (28.9%)
Graduate Degree	227 (47.3%)	152 (45.2%)
*Region*		
Midwest	254 (53.1%)	183 (54.5%)
North East	59 (12.3%)	39 (11.6%)
South	121 (25.3%)	82 (24.4%)
West	42 (8.8%)	31 (9.2%)
Caribbean^a^	2 (0.4%)	1 (0.3%)
*Status of Project*^b^		
Just Started	–	20 (6.0%)
Ongoing	–	174 (51.8%)
Completed	–	142 (42.3%)
	Mean (SD)	
Age (n = 477)	41.6 (14.4)	41.1 (14.4)
Health Literacy—SILS (possible range 1–5)^c^		
Confident with Forms (n = 373)	4.4 (0.8)	4.4 (0.8)
Problems Reading (n = 373)	4.4 (0.9)	4.5 (0.9)
Help Read (n = 373)	4.4 (0.9)	4.4 (0.9)
Numeracy (n = 374)^5^		
SNS ability subscale average (possible range 1 to 5)	3.9 (0.9)	3.9 (0.9)
SNS preference subscale average (possible range 1–6)	4.5 (1.0)	4.6 (1.0)

No statistically significant differences in proportions or means between those who completed survey 4 and those who did not

^a^Virgin Islands (n = 1), Puerto Rico (n = 1)

^b^Asked only on survey 4

^c^Higher scores indicate higher numeracy, health literacy

### REST summary and scores

EP means for the quality scale range from 3.6 to 3.8 (where 1 = poor and 5 = excellent), while means for the quantity scale range from 3.7 to 4.0 (where 1 = never and 5 = always). The mean score on the overall quality version of REST was 3.6 (95% CI 3.5, 3.7) and the overall quantity mean score was 3.9 (95% CI 3.8, 4.0) (Table 2).

**Table 2:** Mean (95% confidence interval) and Cronbach’s alpha for engagement principles—final version of REST

Engagement principle	N items	Quality			Quantity		
		N	Mean (95% CI)	Cronbach’s Alpha	N	Mean (95% CI)	Cronbach’s Alpha
Overall measure	32	224	3.6 (3.5, 3.7)	0.98	234	3.9 (3.8, 4.0)	0.97
EP1: Focus on community perspectives and determinants of health	4	301	3.7 (3.6, 3.8)	0.88	306	3.9 (3.8, 4.0)	0.82
EP2: Partner input is vital	4	306	3.7 (3.6, 3.8)	0.88	311	3.9 (3.8, 4.0)	0.85
EP3: Partnership sustainability to meet goals and objectives	5	291	3.5 (3.4, 3.6)	0.92	298	3.7 (3.6, 3.8)	0.90
EP4: Foster co-learning, capacity building, and co-benefit for all partners	4	313	3.7 (3.6, 3.8)	0.91	324	4.0 (3.9, 4.1)	0.87
EP5: Build on strengths and resources within the community or patient population	3	309	3.8 (3.6, 3.9)	0.88	319	4.0 (3.9, 4.1)	0.83
EP6: Facilitate collaborative, equitable partnerships	4	292	3.6 (3.5, 3.7)	0.90	296	3.9 (3.8, 4.0)	0.87
EP7: Involve all partners in the dissemination process^a^	3	283	3.6 (3.5, 3.7)	0.83	296	3.8 (3.7, 3.9)	0.79
EP8: Build and maintain trust in the partnership	5	301	3.8 (3.6, 3.9)	0.92	304	4.0 (3.9, 4.1)	0.91

^a^Alpha increases slightly to 0.84 (quality scale) and 0.81 (quantity scale) if item 3 removed (All partners have the opportunity to be coauthors when the work is published). However, given the small increase and high alpha this item was retained

When looking at how REST aligns with the categories of stakeholder engagement in research (see Additional file 1: Table S2 for classification information), the range of percent by category ranged widely, as participants were engaged in many different types of projects in our sample across the stakeholder engagement continuum. For the quality scale of REST, average percentages by category of stakeholder engagement ranged from 0 to 100% for outreach and education with a median of 6%; 0–75% for consultation with a median of 9%, 0–81% for cooperation with a median of 25%; 0–75% for collaboration with a median of 41%; and 0–22% for partnership with a median of 3%. For the quantity scale of REST, average percentages ranged from 3 to 84% for outreach and education with a median of 3%; 0–75% for consultation with a median of 9%; 0–78% for cooperation with a median of 22%; 0–75% for collaboration with a median of 47%; and 0–25% for partnership with a median of 6%.

Item specific summary statistics are presented in Table 3. Overall, item means were typically higher for the quantity scale versus the quality scale. The median for all items was 4.0, except for item 7.3 (“All partners have the opportunity to be coauthors when the work is published.”) on the quality scale were the median was 3.0. The item with the highest mean score on both the quantity and quality scales was the same, item 8.4 (“All partners respect the population being served.”). The item with the lowest mean score was also the same on both scales, item 7.3 (“All partners have the opportunity to be coauthors when the work is published.”).

**Table 3:** Item information summary

Item number	Item text	Quantity					Quality				
		Mean	SD	Median	Not applicable—N (%)	Missing—N (%)	Mean	SD	Median	Not applicable—N (%)	Missing—N (%)
1.1	The focus is on problems important to the community	4.10	0.85	4.0	3 (0.9%)	0 (0%)	3.73	1.03	4.0	5 (1.5%)	4 (1.2%)
1.2	All partners look at the data to determine the health problems the community thinks are important	3.80	1.01	4.0	13 (3.9%)	0 (0%)	3.61	1.13	4.0	13 (3.9%)	4 (1.2%)
1.3	The effort incorporates factors (for example housing, transportation, food access, education, employment) that influence health status	3.90	0.96	4.0	18 (5.4%)	0 (0%)	3.62	1.11	4.0	24 (7.1%)	4 (1.2%)
1.4	The focus is on cultural factors that influence health behaviors	3.90	0.96	4.0	15 (4.5%)	0 (0%)	3.66	1.10	4.0	13 (3.9%)	4 (1.2%)
2.1	All partners have the opportunity to share ideas, input, and leadership responsibilities and to share in the determination of the project structure	3.88	0.98	4.0	12 (3.6%)	0 (0%)	3.61	1.11	4.0	13 (3.9%)	5 (1.5%)
2.2	Plans are developed and adjusted to meet the needs and concerns of the community or patient population	3.95	0.96	4.0	12 (3.6%)	0 (0%)	3.70	1.09	4.0	12 (3.6%)	4 (1.2%)
2.3	All partners agree to take on specific tasks according to their comfort, ability, and expertise	3.90	0.96	4.0	10 (3%)	0 (0%)	3.61	1.07	4.0	11 (3.3%)	4 (1.2%)
2.4	All partners assist in establishing roles and related responsibilities for the partnership	3.81	0.96	4.0	8 (2.4%)	0 (0%)	3.51	1.06	4.0	9 (2.7%)	4 (1.2%)
3.1	All partners share updates, progress, strategies, and new ideas regularly	3.82	1.05	4.0	7 (2.1%)	0 (0%)	3.57	1.09	4.0	13 (3.9%)	5 (1.5%)
3.2	A plan is in place for ongoing problem-solving	3.72	1.06	4.0	13 (3.9%)	0 (0%)	3.49	1.16	4.0	13 (3.9%)	4 (1.2%)
3.3	All partners are involved in determining next steps	3.65	1.02	4.0	10 (3%)	0 (0%)	3.41	1.18	3.0	11 (3.3%)	4 (1.2%)
3.4	Community-engaged activities are continued until the goals (as agreed upon by all partners) are achieved	3.75	1.09	4.0	10 (3%)	0 (0%)	3.53	1.16	4.0	10 (3%)	4 (1.2%)
3.5	All partners continue community-engaged activities beyond an initial project, activity, or study	3.59	1.11	4.0	22 (6.5%)	0 (0%)	3.42	1.21	4.0	20 (6%)	4 (1.2%)
4.1	All partners have a variety of opportunities to gain new skills or knowledge from their involvement	3.80	0.95	4.0	4 (1.2%)	0 (0%)	3.56	1.09	4.0	8 (2.4%)	5 (1.5%)
4.2	All partners are encouraged to learn from each other	4.02	0.99	4.0	4 (1.2%)	2 (0.6%)	3.68	1.11	4.0	6 (1.8%)	6 (1.8%)
4.3	The partnership adds value to the work of all partners	4.02	0.96	4.0	4 (1.2%)	1 (0.3%)	3.76	1.08	4.0	5 (1.5%)	5 (1.5%)
4.4	All partners share resources to increase ability to address the problem of interest	3.80	1.01	4.0	7 (2.1%)	1 (0.3%)	3.65	1.09	4.0	12 (3.6%)	5 (1.5%)
5.1	The team builds on strengths and resources within the community or patient population	3.88	1.05	4.0	9 (2.7%)	1 (0.3%)	3.61	1.11	4.0	11 (3.3%)	6 (1.8%)
5.2	The team works with existing community groups and organizations	4.08	0.98	4.0	6 (1.8%)	1 (0.3%)	3.76	1.13	4.0	8 (2.4%)	5 (1.5%)
5.3	The team includes representation from the local community or patient population	3.91	0.99	4.0	10 (3%)	1 (0.3%)	3.68	1.11	4.0	16 (4.8%)	5 (1.5%)
6.1	Fair processes have been established to manage conflict or disagreements	3.71	1.06	4.0	21 (6.3%)	0 (0%)	3.48	1.14	4.0	24 (7.1%)	5 (1.5%)
6.2	All partners ideas are treated with openness and respect	4.08	0.91	4.0	3 (0.9%)	0 (0%)	3.69	1.09	4.0	7 (2.1%)	5 (1.5%)
6.3	All partners agree on the timeline for making shared decisions about the project	3.83	0.92	4.0	10 (3%)	1 (0.3%)	3.54	1.07	4.0	13 (3.9%)	5 (1.5%)
6.4	All partners agree on ownership of data for publications and presentations	3.78	1.07	4.0	25 (7.4%)	0 (0%)	3.51	1.14	4.0	22 (6.5%)	4 (1.2%)
7.1	All partners can use knowledge generated from the partnership	4.07	0.97	4.0	7 (2.1%)	0 (0%)	3.75	1.11	4.0	8 (2.4%)	4 (1.2%)
7.2	All interested partners are involved in activities related to sharing results	3.76	1.09	4.0	9 (2.7%)	0 (0%)	3.63	1.17	4.0	10 (3%)	5 (1.5%)
7.3	All partners have the opportunity to be coauthors when the work is published	3.47	1.19	4.0	36 (10.7%)	0 (0%)	3.20	1.32	3.0	41 (12.2%)	4 (1.2%)
8.1	The partnerships processes support trust among all partners	3.89	0.95	4.0	9 (2.7%)	0 (0%)	3.65	1.11	4.0	9 (2.7%)	4 (1.2%)
8.2	All partners are confident that they will receive credit for their contributions to the partnership	3.71	1.13	4.0	18 (5.4%)	0 (0%)	3.46	1.25	4.0	19 (5.7%)	4 (1.2%)
8.3	Mutual respect exists among all partners	3.96	0.99	4.0	6 (1.8%)	0 (0%)	3.75	1.07	4.0	7 (2.1%)	4 (1.2%)
8.4	All partners respect the population being served	4.12	0.98	4.0	8 (2.4%)	0 (0%)	3.90	1.08	4.0	8 (2.4%)	4 (1.2%)
8.5	All partners understand the culture of the organizations and community(ies) involved in the partnership	3.78	1.02	4.0	7 (2.1%)	0 (0%)	3.67	1.12	4.0	9 (2.7%)	4 (1.2%)

Six (19%) items had a large amount of not applicable responses (> 5%). Items that met this criteria were consistent on both the quality and quantity scales and included items:

- 1.3—“The effort incorporates factors (for example housing, transportation, food access, education, employment) that influence health status.”
- 3.5—“All partners continue community-engaged activities beyond an initial project, activity, or study.”
- 6.1—“Fair processes have been established to manage conflict or disagreements.”
- 6.4—“All partners agree on ownership of data for publications and presentations.”
- 7.3—“All partners have the opportunity to be coauthors when the work is published.”
- 8.2—“All partners are confident that they will receive credit for their contributions to the partnership.”

### Internal consistency

Results on the final comprehensive version of REST (from survey four) showed strong internal consistency among the EPs for both the quality (Cronbach’s alpha range: 0.83 to 0.92) and quantity (Cronbach’s alpha range: 0.79–0.91) versions of the measure (Table 2). For EP 7 (*Involve all partners in the dissemination process*), results showed a slight increase in alpha if item 7.3 (*All partners have the opportunity to be coauthors when the work is published*) was removed. The alpha increased slightly to 0.84 from 0.83 for the quality version, and 0.81 from 0.79 for the quantity version. However, given the slight improvements with the deletion of this item it was retained in the comprehensive REST.

### Convergent validity

REST was significantly correlated with several of the comparison measures we used (Table 4). REST showed a statistically significant, but negligible positive correlation with Mainous trust in medical researchers scale (quantity only: r = 0.12, *p* = 0.03) [46], Hall trust in medical researchers (quality: r = 0.18, *p* < 0.001; quantity: r = 0.21, *p* < 0.001) [47], the CERI (quality: r = 0.19, *p* = 0.001; quantity: r = 0.25, *p* < 0.001)[11], and PSAT drawbacks dimension (quality: r = − 0.21, *p* < 0.001; quantity: r = − 0.26, *p* < 0.001) [49, 50]. There was a negligible and insignificant correlation between REST and each of the single item literacy screeners and a negligible significant correlation between the REST and the subjective numeracy ability (quality: r = 0.11, *p* = 0.04; quantity: r = 0.11, *p* = 0.05) and preferences (quality: r = 0.12, *p* = 0.03; quantity: r = 0.12, *p* = 0.03) subscales [43–45].

**Table 4:** Comprehensive version of REST convergent validity with other measures

Other measures	Quality			Quantity		
	N	Spearman’s R	*P* value	N	Spearman’s R	*P* value
Medical Mistrust	322	0.11 (negligible)	0.05	325	0.12 (negligible)	0.03
Trust in Medical Researchers	322	0.18 (negligible)	< 0.001	324	0.21 (negligible)	< 0.001
Partnership Assessment in community-based Research (PAIR) Measure	322	0.34 (low)	< 0.001	325	0.44 (low)	< 0.001
Kagan Survey of community engagement	319	0.50 (moderate)	< 0.001	322	0.56 (moderate)	< 0.001
Community Engagement in Research Index (CERI)	320	0.19 (negligible)	0.001	323	0.25 (negligible)	< 0.001
Coalition Self-Assessment Survey (CSAS)—Trust^a^	323	0.40 (low)	< 0.001	328	0.42 (low)	< 0.001
*Partnership self-assessment tool (PSAT)*						
PSAT—Synergy	325	0.61 (moderate)	< 0.001	328	0.62 (moderate)	< 0.001
PSAT—Satisfaction	324	0.61 (moderate)	< 0.001	327	0.65 (moderate)	< 0.001
PSAT—Leadership	323	0.69 (moderate)	< 0.001	326	0.69 (moderate)	< 0.001
PSAT—Efficiency	323	0.62 (moderate)	< 0.001	326	0.59 (moderate)	< 0.001
PSAT—Administration/Management	323	0.63 (moderate)	< 0.001	326	0.64 (moderate)	< 0.001
PSAT—Non-Financial Resources	322	0.47 (low)	< 0.001	325	0.52 (moderate)	< 0.001
PSAT—Financial and Other Capital Resources	320	0.34 (low)	< 0.001	323	0.32 (low)	< 0.001
PSAT—Decision Making	325	0.51 (moderate)	< 0.001	328	0.51 (moderate)	< 0.001
PSAT—Benefits	324	0.33 (low)	< 0.001	327	0.41 (low)	< 0.001
PSAT—Drawbacks	324	− 0.21 (negligible)	< 0.001	327	− 0.26 (negligible)	< 0.001
PSAT—Comparing Benefits and Drawbacks	323	0.39 (low)	< 0.001	326	0.42 (low)	< 0.001
Wilder Collaboration	325	0.54 (moderate)	< 0.001	328	0.54 (moderate)	< 0.001

^a^Correlation with EP8 (Build and maintain trust in the partnership) only

The REST has a low positive correlation with the PAIR (quality: r = 0.34, *p* < 0.001; quantity: r = 0.44, *p* < 0.001) [40], PSAT non-financial resources dimension (quality only: r = 0.47, *p* < 0.001), PSAT benefits dimension (quality: r = 0.33, *p* < 0.001; quantity: r = 0.41, *p* < 0.001), and PSAT comparing benefits and drawbacks dimension (quality: r = 0.39, *p* < 0.001; quantity: r = 0.42, *p* < 0.001) [49, 50]. REST EP8 (Build and maintain trust in the partnership) showed a low positive correlation with the trust measure of the CSAS (quality: r = 0.40, *p* < 0.001; quantity: r = 0.42, *p* < 0.001) [35].

The REST has a moderate correlation with the Kagan et al. measure (quality: r = 0.50, *p* < 0.001; quantity: r = 0.56, *p* < 0.001) [34] and the Wilder collaboration inventory (quality: r = 0.54, *p* < 0.001; quantity: r = 0.54, *p* < 0.001) [51, 52]. REST also showed a moderate correlation with seven dimensions of the PSAT: synergy dimension (quality: r = 0.61, *p* < 0.001; quantity: r = 0.62, *p* < 0.001), satisfaction dimension (quality: r = 0.61, *p* < 0.001; quantity: r = 0.65, *p* < 0.001), non-financial resources dimension (quantity only: r = 0.52, *p* < 0.001), leadership dimension (quality: r = 0.69, *p* < 0.001; quantity: r = 0.69, *p* < 0.001), efficiency dimension (quality: r = 0.62, *p* < 0.001; quantity: r = 0.59, *p* < 0.001), administration/management dimension (quality: r = 0.63, *p* < 0.001; quantity: r = 0.64, *p* < 0.001), and the decision making dimension (quality: r = 0.51, *p* < 0.001; quantity: r = 0.51, *p* < 0.001) [49, 50, 53].

While the statistically significant correlations show the measures are correlated (as theoretically hypothesized), the levels of correlation were negligible, low or moderate.

## Discussion

We examined the internal consistency and construct validity of the REST. Given the lack of a gold standard measure of stakeholder engagement in research, we calculated the correlation (convergent validity) with other theoretically related constructs (e.g., partnership, collaboration, community engagement, trust, and mistrust). We found statistically significant correlations (negligible, low, moderate) with other measures theoretically associated with stakeholder engagement. However, the lack of high correlation with any of the existing measures suggests the REST is measuring a different construct (perceived stakeholder engagement in research) than these existing measures. Together the results suggest the REST is a valid (research engagement construct) and reliable (internally consistent) tool to assess research engagement of non-academic stakeholders in research. Valid and reliable tools to assess research engagement are necessary to examine the impact of stakeholder engagement on the scientific process and scientific discovery and move the field of stakeholder engagement from best practices and lessons learned to evidence-based approaches based on empirical data and rigorous scientific study designs.

### Strengths, limitations, and future directions

Our study findings should be considered in the context of several limitations. First, recruitment delays caused the study design to change, and we ended up recruiting throughout the entire study period versus recruiting all participants before survey one, then consecutively releasing surveys two, three and four to all participants at once. This resulted in some participants doing surveys closer together, while some did them further apart. However, only 31 participants (6%) did the surveys out of order, while 456 (94%) did the surveys in order. Demographic characteristics did not differ between these participants. Second, timing of surveys could have had an effect on those involved in ongoing projects. On survey four, we asked participants to classify the status of their project (just started, ongoing, completed). Of the 336 that completed survey four, 20 (6%) indicated that the project had just started, 174 (52%) indicated that the project was ongoing, and 142 (42%) indicated that the project had been completed (Table 1). Participants whose project was ongoing or had just started may have had changes in the level of engagement across the four surveys, whereas those with complete projects may not have had changes in the level of engagement across the four surveys.

Third, we had a large portion of participants lost to follow up, leading to a smaller sample size for survey four as compared to the number of participants who completed consent. Attrition rate for this study was 31% (151 lost to follow up by survey four) most of which occurred from participants not completing any of the surveys (n = 94; 19%). Among participants that completed one survey (n = 393), 85% completed the final longitudinal survey. While 80% follow-up has been stated as a cut-off rate for acceptable loss to follow-up [54], a systematic review of longitudinal studies found an average retention rate of 74% (standard deviation = 20%), a rate that remained consistent independent of the duration of the study or study type [55]. Studies on the impact of attrition in longitudinal research generally suggest that a 25–30% loss to follow up is acceptable [56], with the impact of further attrition dependent on the extent to which the data is missing at random (acceptable results with up to 25–60% loss to follow-up) or missing not at random (bias present at 30% loss to follow-up) [57, 58].

We also had a higher percent of missing responses on the final version of REST (survey four). This was due primarily to the addition of a ‘not applicable’ response option. This was indicated by the low number of missing responses other than “not applicable” responses among all items (Table 3). Due to attrition and missing data some of the analyses are based on samples of size 224 (67% of the analytic sample). However, we conducted a sensitivity analysis to compare complete case data and data including missingness and found the results to be similar. Finally, REST is currently only available in English, and we were unable to estimate the time to complete. We have time to complete the entire survey, calculated as the finished time minus the starting time. However, the survey had additional questions and participants could start and stop the survey and return later to complete. Excluding those that took more than 30 min to complete, the mean time to completion for the survey is 14 min (median = 13 min) based on this we estimate the REST takes less than 10 min to complete.

Despite these limitations, REST and our study have several strengths. The REST was developed through a stakeholder engaged process from a community-academic partnership (Disparities Elimination Advisory Committee) and was validated using input from stakeholders (e.g., patients and their families, clinicians, health systems, policy makers, community organizations, advocacy groups). REST is flexible and a general tool that can be used across a variety of project types, stages, and stakeholder groups (e.g., community advisory boards, patients, community members, health departments, health care organizations) [59]. REST is easy to administer via online web-survey and also shows potential to be completed via a paper-based survey. The REST is disease, demographic group (e.g., gender, race, age), and stakeholder group (e.g., community advisory boards, patients, community members, health departments, health care organizations) agnostic to allow for use across a broad range of community engaged research activities [59]. The REST was designed to fill a gap due to the dearth of existing measures on stakeholder engagement in research. Measures that assess the perceived level of engagement of non-academic research partners across a broad array of engagement activities, research projects, diseases, health outcomes, and populations are necessary to build an evidence-base for stakeholder engagement by determining the quantity and quality of engagement necessary for successful outcomes.

## Conclusions

The REST is a tool that examines how stakeholders (e.g., patients and their families, clinicians, health systems, policy makers, community organizations, advocacy groups) understand and experience their engagement in research projects. In the future, REST should be developed and validated in languages other than English (e.g., Spanish and Mandarin). We do not believe direct translation is appropriate but believe we have developed an approach that can be adapted to other languages. In an implementation study we demonstrate the ability of the REST to measure engagement across a broad array of projects with different levels of engagement [59]. However, the REST should also be examined longitudinally in projects over time to examine test re-test reliability and to see how sensitive REST is to changes in engagement over time. This would allow for the examination of the quantity and quality of engagement necessary to move partnerships along the engagement continuum. Tools that assess stakeholder engagement are necessary for the empirical examination of the influence of engagement on the types of research, questions addressed, service improvement, scientific process, and scientific discovery.

## Supplementary Information

**Additional file 1: Table S1.** Screening Questions on Involvement in Community Engagement in Research - A three item screening survey used to screen potential participants for the longitudinal surveys. **Table S2.** Classification of Comprehensive (32-item) REST Items by Categories of Engagement - Describes the REST scoring approach that is aligned with the five levels of engagement: Outreach and Education, Consultation, Cooperation, Collaboration, and Partnership.

## Data Availability

The datasets used and/or analyzed during the current study are available from the corresponding author on reasonable request and regulatory (IRB) approval.

## Abbreviations

REST: Research Engagement Survey Tool
PI(s): Principal investigator(s)
EP(s): Engagement principle(s)
CTSA: Clinical and Translational Science Awards
PAIR: Partnership Assessment In community-based Research
CERI: Community engagement research index
CSAS: Coalition self-assessment survey
CCPH: Community campus partnerships for health
PSAT: Partnership self-assessment tool
CI: Confidence interval
GED: General Education Develop test (high school equivalent)

## References

[1] Huzzard T (2021). Achieving impact: exploring the challenge of stakeholder engagement. Eur J Work Organ Psychol [Internet].

[2] Goodman MS, Sanders Thompson VL (2017). The science of stakeholder engagement in research: classification, implementation, and evaluation. Transl Behav Med [Internet].

[3] Holzer JK, Ellis L, Merritt MW (2014). Why we need community engagement in medical research. J Investig Med [Internet].

[4] Cyril S, Smith BJ, Possamai-Inesedy A, Renzaho AMN (2015). Exploring the role of community engagement in improving the health of disadvantaged populations: a systematic review. Glob Health Action [Internet].

[5] Boote J, Telford R, Cooper C (2002). Consumer involvement in health research: a review and research agenda. Health Policy (New York) [Internet].

[6] O’Mara-Eves A, Brunton G, Oliver S, Kavanagh J, Jamal F, Thomas J (2015). The effectiveness of community engagement in public health interventions for disadvantaged groups: a meta-analysis. BMC Public Health [Internet].

[7] Milton B, Attree P, French B, Povall S, Whitehead M, Popay J (2012). The impact of community engagement on health and social outcomes: a systematic review. Community Dev J.

[8] Schulz AJ, Israel BA, Lantz P (2003). Instrument for evaluating dimensions of group dynamics within community-based participatory research partnerships. Eval Program Plann.

[9] Lantz PM, Viruell-Fuentes E, Israel BA, Softley D, Guzman R (2001). Can communities and academia work together on public health research? Evaluation results from a community-based participatory research partnership in Detroit. J Urban Heal [Internet].

[10] Israel BA, Schulz AJ, Parker EA, Becker AB (1998). Review of community-based research: assessing partnership approaches to improve public health [Internet]. Annu Rev Public Health.

[11] Khodyakov D, Stockdale S, Jones A, Mango J, Jones F, Lizaola E (2013). On measuring community participation in research. Health Educ Behav [Internet].

[12] Francisco VT, Paine AL, Fawcett SB (1993). A methodology for monitoring and evaluating community health coalitions. Health Educ Res [Internet].

[13] Goodman MS, Sanders Thompson VL, Arroyo Johnson C, Gennarelli R, Drake BF, Witherspoon M (2017). Evaluating community engagement in research: quantitative measure development. J Community Psychol [Internet].

[14] Bowen DJ, Hyams T, Goodman M, West KM, Harris-Wai J, Yu JH (2017). Systematic review of quantitative measures of stakeholder engagement. Clin Transl Sci.

[15] Sanders Thompson VL, Ackermann N, Bauer KL, Bowen DJ, Goodman MS (2020). Strategies of community engagement in research: definitions and classifications. Transl Behav Med [Internet].

[16] Goodman MS, Ackermann N, Bowen DJ, Thompson V (2019). Content validation of a quantitative stakeholder engagement measure. J Community Psychol [Internet].

[17] Arroyo-Johnson C, Allen ML, Colditz GA, Hurtado GA, Davey CS, Thompson VLS (2015). A tale of two community networks program centers: operationalizing and assessing CBPR principles and evaluating partnership outcomes. Prog Community Heal Partners Res Educ Action [Internet].

[18] Thompson VLS, Drake B, James AS, Norfolk M, Goodman M, Ashford L (2014). A community coalition to address cancer disparities: transitions, successes and challenges. J Cancer Educ [Internet].

[19] Israel BA, Schulz AJ, Parker EA, Becker A (1998). Review of community-based research: assessing partnership approaches to improve public health. Annu Rev Public Health [Internet].

[20] McCloskey DJ, McDonald MA, Cook J, Heurtin-Roberts S, Updegrove S, Sampson D (2012). Community engagement : definitions and organizing concepts from the literature. Prince Community Engagem.

[21] Khodyakov D, Stockdale S, Jones F, Ohito E, Jones A, Lizaola E (2011). An exploration of the effect of community engagement in research on perceived outcomes of partnered mental health services projects. Soc Ment Health [Internet].

[22] Wallerstein NB, Duran B (2006). Using community-based participatory research to address health disparities. Health Promot Pract.

[23] Nueces DL, Hacker K, DiGirolamo A, Hicks S, De Las-Nueces D, Hicks LS (2012). A systematic review of community-based participatory research to enhance clinical trials in racial and ethnic minority groups. Health Serv Res [Internet].

[24] Burke JG, Hess S, Hoffmann K, Guizzetti L, Loy E, Gielen A (2013). Translating community-based participatory research principles into practice. Prog Community Heal Partnerships Res Educ Action [Internet].

[25] Israel BA, Schulz AJ, Parker EA, Becker AB, Allen AJ, Guzman JR. Critical issues in developing and following CBPR principles. Community Based Particip Res Health Process Outcomes; 2008;47–66.

[26] Butterfoss FD, Francisco VT (2004). Evaluating community partnerships and coalitions with practitioners in mind. Heal Promot Pract [Internet].

[27] Clinical and Translational Science Awards Consortium Community Engagement Key Function Committee Task Force on the Principles of Community Engagement. Principles of Community Engagement [Internet]. NIH Publication No. 11-7782; 2011. http://www.atsdr.cdc.gov/communityengagement/.

[28] Ahmed SM, Palermo A-GS (2010). Community engagement in research: frameworks for education and peer review. Am J Public Health [Internet].

[29] Butterfoss FD, Goodman RM, Wandersman A (1996). Community coalitions for prevention and health promotion: factors predicting satisfaction, participation, and planning. Health Educ Q.

[30] Goodman MS, Ackermann N, Bowen DJ, Panel D, Thompson VS (2020). Reaching consensus on principles of stakeholder engagement in research. Prog Community Health Partnersh.

[31] Thompson VLS, Leahy N, Ackermann N, Bowen DJ, Goodman MS (2020). Community partners’ responses to items assessing stakeholder engagement: cognitive response testing in measure development. PLoS ONE.

[32] Hamilton CB, Hoens AM, McQuitty S, McKinnon AM, English K, Backman CL (2018). Development and pre-testing of the Patient Engagement In Research Scale (PEIRS) to assess the quality of engagement from a patient perspective. PLoS ONE.

[33] Khodyakov D, Stockdale S, Jones A, Mango J, Jones F, Lizaola E (2013). On measuring community participation in research. Heal Educ Behav [Internet].

[34] Kagan JM, Rosas SR, Siskind RL, Campbell RD, Gondwe D, Munroe D (2012). Community-researcher partnerships at NIAID HIV/AIDS clinical trials sites: insights for evaluation & enhancement. Prog Community Health Partnersh.

[35] Peterson JW, Lachance LL, Butterfoss FD, Houle CR, Nicholas EA, Gilmore LA (2006). Engaging the community in coalition efforts to address childhood asthma. Health Promot Pract [Internet].

[36] Streiner DL, Norman GR, Cairney J (2015). Health measurement scales: a practical guide to their development and use.

[37] Barkto J, Bartko JJ (1966). The intraclass correlation coefficient as a measure of reliability. Psychol Rep.

[38] Iacobucci D, Duhachek A (2003). Advancing alpha: measuring reliability with confidence. J Consum Psychol [Internet].

[39] Nunnally J, Bernstein I. Psychometric theory. 1994 [cited 2021 May 8]. http://vlib.kmu.ac.ir/kmu/handle/kmu/84743.

[40] Arora PG, Krumholz LS, Guerra T, Leff SS (2015). Measuring community-based participatory research partnerships: the initial development of an assessment instrument. Prog Community Heal Partnerships Res Educ Action [Internet].

[41] Barge S, Gehlbach H (2012). Using the theory of satisficing to evaluate the quality of survey data. Res High Educ.

[42] Leiner DJ (2019). Too fast, too straight, too weird: non-reactive indicators for meaningless data in internet surveys. Surv Res Methods [Internet].

[43] Chew LD, Griffin JM, Partin MR, Noorbaloochi S, Grill JP, Snyder A (2008). Validation of screening questions for limited health literacy in a large VA outpatient population. J Gen Intern Med.

[44] Chew LD, Bradley KA, Boyko EJ (2004). Brief questions to identify patients with inadequate health literacy. Fam Med.

[45] Fagerlin A, Zikmund-Fisher BJ, Ubel PA, Jankovic A, Derry HA, Smith DM (2007). Measuring numeracy without a math test: development of the Subjective Numeracy Scale (SNS). Med Decis Mak.

[46] Mainous AG, Smith DW, Geesey ME, Tilley BC (2006). Development of a measure to assess patient trust in medical researchers. Ann Fam Med [Internet].

[47] Hall M, Camacho F, Lawlor JS, Depuy V, Sugarman J, Weinfurt K (2006). Measuring trust in medical researchers. Med Care.

[48] Bell-Eikins JB (2002). A case study of a successful community-campus partnership: changing the environment through collaboration.

[49] Center for the Advancement of Collaborative Strategies in Health. Partnership self-assessment tool: questionnaire [Internet]. 2002. https://atrium.lib.uoguelph.ca/xmlui/bitstream/handle/10214/3129/Partnership_Self-Assessment_Tool-Questionnaire_complete.pdf?sequence=1&isAllowed=y.

[50] National Collaborating Center for Methods and Tools. Partnership evaluation: the partnership self-assessment tool [Internet]. 2008 [cited 2019 Jan 8]. https://www.nccmt.ca/knowledge-repositories/search/10.

[51] Mattessich PW, Murray-Close M, Monsey BR, Wilder Research Center (2001). Collaboration: what makes it work, a review of research literature on factors influencing successful collaboration.

[52] Derose K, Beatty A, Jackson C (2004). Evaluation of community voices Miami: affecting health policy for the uninsured [Internet].

[53] Luke DA, Calhoun A, Robichaux CB, Elliott MB, Moreland-Russell S (2014). The program sustainability assessment tool: a new instrument for public health programs. Prev Chronic Dis [Internet].

[54] Von Elm E, Altman DG, Egger M, Pocock SJ, Gøtzsche C, Vandenbroucke JP (2007). Policy and practice the strengthening the reporting of observational studies in epidemiology (STROBE) statement: guidelines for reporting observational studies*. Bull World Health Organ.

[55] Teague S, Youssef GJ, Macdonald JA, Sciberras E, Shatte A, Fuller-Tyszkiewicz M (2018). Retention strategies in longitudinal cohort studies: a systematic review and meta-analysis. BMC Med Res Methodol [Internet].

[56] der Wiel AB, van Exel E, de Craen AJ, Gussekloo J, Lagaay A, Knook D (2002). A high response is not essential to prevent selection bias. J Clin Epidemiol [Internet].

[57] Kristman V, Manno M, Côté P (2003). Loss to follow-up in cohort studies: how much is too much?. Eur J Epidemiol [Internet].

[58] Gustavson K, von Soest T, Karevold E, Røysamb E (2012). Attrition and generalizability in longitudinal studies: findings from a 15-year population-based study and a Monte Carlo simulation study. BMC Public Health [Internet].

[59] Bowen DJ, Ackermann N, Thompson VS, Nederveld A, Goodman M (2022). A study examining the usefulness of a new measure of research engagement. J Gen Intern Med [Internet].

