# Supplementary material for: Construct validation of the Research Engagement Survey Tool (REST)
Source: Res Involv Engagem. 2022 Jun 16;8:26. doi: 10.1186/s40900-022-00360-y (PMC9204858; doi:10.1186/s40900-022-00360-y)
Supplement: Supplementary file 1 — Additional file 1: Table S1. Screening Questions on Involvement in Community Engagement in Research - A three item screening survey used to screen potential participants for the longitudinal surveys. Table S2. Classification of Comprehensive (32-item) REST Items by Categories of Engagement - Describes the REST scoring approach that is aligned with the five levels of engagement: Outreach and Education, Consultation, Cooperation, Collaboration, and Partnership. [file 40900_2022_360_MOESM1_ESM.docx]

**Additional file 1**

**Table S1. Screening Questions on Involvement in Community Engagement in Research**

1.) How many community engaged research projects have you participated in? _____________

2.) Have you served as a stakeholder on a community engaged research project or community-academic partnership?

1. Yes
2. No

***Participant must either answer 1 or greater to question 1 OR ‘yes’ to question 2 to be ELIGIBLE*

3.) Please check all the research activities that you have been involved with either as “consultants” or “active participants.” If you have not been involved with any activities listed below, please leave this question blank.

- Grant proposal writing
- Background research
- Choosing research methods
- Developing sampling procedures
- Recruiting study participants
- Implementing the intervention
- Designing interview and/or survey questions
- Collecting primary data
- Analyzing collected data
- Interpreting study findings
- Writing reports and journal articles
- Giving presentations at meetings and conferences
- Served on a community advisory board (CAB)

***If participant does not select any of the listed above for question 3, they are INELIGIBLE*

| **Table S2.** Classification of Comprehensive (32-item) REST Items by Categories of Engagement | | | | | | |
| --- | --- | --- | --- | --- | --- | --- |
| **Engagement Principle (EP)** | **Starting Level of Engagement – by EP** | **Likert Response Options** | | | | |
|  |  | **Quality** | | | | |
|  |  | **Poor** | **Fair** | **Good** | **Very Good** | **Excellent** |
|  |  | **Quantity** | | | | |
|  |  | **Never** | **Rarely** | **Sometimes** | **Often** | **Always** |
| **EP1. Focus on community perspectives and determinants of health** | **Collaboration** | -- | -- | -- | -- | -- |
| 1.1: The focus is on problems important to the community. | Collaboration | O&E | Con | Coop | Col | Col |
| 1.2: All partners look at the data to determine the health problems the community thinks are important. | Collaboration | O&E | Con | Coop | Col | Col |
| 1.3: The effort incorporates factors (for example—housing, transportation, food access, education, employment) that influence health status. | Outreach & Education | O&E | O&E | O&E | O&E | O&E |
| 1.4: The focus is on cultural factors that influence health behaviors. | Consultation (quantity) Cooperation (quality) | O&E | O&E | O&E | Con | Coop (quality); Con (quantity) |
| **EP2. Partner input is vital** | **Collaboration** | **--** | **--** | **--** | **--** | **--** |
| 2.1: All partners have the opportunity to share ideas, input, and leadership responsibilities and to share in the determination of the project structure. | Collaboration | O&E | Con | Coop | Col | Col |
| 2.2: Plans are developed and adjusted to meet the needs and concerns of the community or patient population. | Collaboration | O&E | Con | Coop | Col | Col |
| 2.3: All partners agree to take on specific tasks according to their comfort, ability, and expertise. | Cooperation | O&E | O&E | Con | Coop | Coop |
| 2.4: All partners assist in establishing roles and related responsibilities for the partnership. | Collaboration | O&E | Con | Coop | Col | Col |
| **EP3. Partnership sustainability to meet goals and objectives** | **Partnership** | -- | -- | -- | -- | -- |
| 3.1: All partners share updates, progress, strategies, and new ideas regularly. | Cooperation | O&E | O&E | Con | Coop | Coop |
| 3.2: A plan is in place for ongoing problem-solving. | Partnership | O&E | Con | Coop | Col | Part |
| 3.3: All partners are involved in determining next steps. | Collaboration | O&E | Con | Coop | Col | Col |
| 3.4: Community-engaged activities are continued until the goals (as agreed upon by all partners) are achieved. | Partnership | O&E | Con | Coop | Col | Part |
| 3.5: All partners continue community-engaged activities beyond an initial project, activity, or study. | Partnership | O&E | Con | Coop | Col | Part |
| **EP4. Foster co-learning, capacity building, and co-benefit for all partners** | **Collaboration** | -- | -- | -- | -- | -- |
| 4.1: All partners have a variety of opportunities to gain new skills or knowledge from their involvement. | Collaboration | O&E | Con | Coop | Col | Col |
| 4.2: All partners are encouraged to learn from each other. | Collaboration | O&E | Con | Coop | Col | Col |
| 4.3: The partnership adds value to the work of all partners. | Collaboration | O&E | Con | Coop | Col | Col |
| 4.4: All partners share resources to increase ability to address the problem of interest. | Collaboration | O&E | Con | Coop | Col | Col |
| **EP5. Build on strengths and resources within the community or patient population** | **Cooperation** | **--** | **--** | **--** | **--** | **--** |
| 5.1: The team builds on strengths and resources within the community or patient population. | Consultation | O&E | O&E | O&E | Con | Con |
| 5.2: The team works with existing community groups and organizations. | Cooperation | O&E | O&E | Cons | Coop | Coop |
| 5.3: The team includes representation from the local community or patient population. | Cooperation | O&E | O&E | Con | Coop | Coop |
| **EP6. Facilitate collaborative, equitable partnerships** | **Partnership** | -- | -- | -- | -- | -- |
| 6.1: Fair processes have been established to manage conflict or disagreements. | Partnership | O&E | Con | Coop | Col | Part |
| 6.2: All partners’ ideas are treated with openness and respect. | Collaboration | O&E | Con | Coop | Col | Col |
| 6.3: All partners agree on the timeline for making shared decisions about the project. | Collaboration | O&E | Con | Coop | Col | Col |
| 6.4: All partners agree on ownership of data for publications and presentations. | Collaboration | O&E | Con | Coop | Col | Col |
| **EP7. Involve all partners in the dissemination process** | **Partnership** | -- | -- | -- | -- | -- |
| 7.1: All partners can use knowledge generated from the partnership. | Col (quality)  Part (quantity) | O&E | Con | Coop | Col | Col (quality);  Part (quantity) |
| 7.2: All interested partners are involved in activities related to sharing results. | Cooperation | O&E | O&E | Con | Coop | Coop |
| 7.3: All partners have the opportunity to be coauthors when the work is published. | Partnership | O&E | Con | Coop | Col | Part |
| **EP8. Build and maintain trust in the partnership** | **Partnership** | -- | -- | -- | -- | -- |
| 8.1: The partnership’s processes support trust among all partners. | Partnership | O&E | Con | Coop | Col | Part |
| 8.2: All partners are confident that they will receive credit for their contributions to the partnership. | Collaboration | O&E | Con | Coop | Col | Col |
| 8.3: Mutual respect exists among all partners. | Collaboration | O&E | Con | Coop | Col | Col |
| 8.4: All partners respect the population being served. | Collaboration | O&E | Con | Coop | Col | Col |
| 8.5: All partners understand the culture of the organizations and community(ies) involved in the partnership. | Partnership | O&E | Con | Coop | Col | Part |

Abbreviations: EP: engagement principle, O&E= Outreach and Education, Con=Consultation, Coop=Cooperation, Col=Collaboration, Part=Partnership, REST=Research Engagement Survey Tool.
